# The Hospital Saturday Fund's Method of Distribution

**Published:** 1897-04-10

**Authors:** 


### THE HOSPITAL SATURDAY FUND'S METHOD OF DISTRIBUTION.

We have received a letter from Mr. Acland in regard to an editorial comment under the above heading in our issue of April 3rd, which we are unable to publish until next week owing to pressure on our space.

